# Patients with dorsolateral prefrontal cortex lesions are capable of discriminatory threat learning but appear impaired in cognitive regulation of subjective fear

**DOI:** 10.1093/scan/nsz039

**Published:** 2019-05-23

**Authors:** Marijn C W Kroes, Joseph E Dunsmoor, Mathew Hakimi, Sofie Oosterwaal, NYU PROSPEC collaboration, Michael R Meager, Elizabeth A Phelps

**Affiliations:** 1 Department of Psychology, New York University, New York, NY 10003, USA; 2 Center for Neural Science, New York University, New York, NY 10003, USA; 3 Donders Institute for Brain, Cognition, and Behaviour, Radboud University Nijmegen Medical Center, 6500 HB Nijmegen, Netherlands; 4 Department of Psychiatry, University of Texas at Austin, Austin, TX 78712, USA; 5 Department of Neurology, New York University School of Medicine, New York, NY 10016, USA; 6 Department of Psychology, Harvard, Cambridge, MA 02138, USA

**Keywords:** emotion, cognition, regulation, prefrontal cortex, lesions, patients

## Abstract

Humans are able to cognitively regulate emotions by changing their thoughts. Neuroimaging studies show correlations between dorsolateral prefrontal cortex (dlPFC) activity and cognitive regulation of emotions. Here our objective was to investigate whether dlPFC damage is associated with impaired cognitive regulation of emotion. We therefore tested the ability of patients with dlPFC lesions (*N* = 6) and matched control participants (*N* = 19) to utilize a laboratory version of cognitive regulation training (CRT) to regulate subjective fear and autonomic threat responses following Pavlovian threat conditioning. We found that patients with dlPFC lesions were able to acquire conditioned threat but seemed impaired in their ability to utilize CRT to cognitively regulate subjective fear to a threatening stimulus. Despite inclusion of a limited number of lesion patients, our results suggest that the dlPFC is important for the cognitive regulation of subjective fear.

## Introduction

Emotional learning and regulation is critical to adaptive behaviour. Threat (‘fear’) conditioning has long been used to study emotional learning and regulation in humans and non-human animals. Both humans and non-human animals can learn to regulate threat responses via extinction learning, and studies show that humans also have the ability to change their emotions by changing their thoughts (Gross and Levenson, 1997; Ochsner and Gross, 2005; Delgado *et al.,* 2008). Such cognitive regulation allows us to reinterpret stimuli or events and regulate our emotional subjective feelings and autonomic responses (Gross and Levenson, 1997; Ochsner and Gross, 2005; Delgado *et al.,* 2008). Cognitive regulation (or restructuring) is also a common component of cognitive behavioural therapy (CBT). In CBT, cognitive restructuring is used to teach patients to reinterpret negative stimuli by identifying irrational thoughts and beliefs and changing them using adaptive coping mechanisms that reduce emotional responses when the stimuli are encountered again (Beck and Dozois, 2011). CBT is an effective treatment for a range of stress and anxiety disorders. However, CBT does not work for all patients and many patients experience a return of symptoms even after initially successful treatment.

Neuroimaging studies have revealed correlations between cognitive regulation of emotions and neural activity in the dorsolateral prefrontal cortex (dlPFC) (Beauregard *et al.,* 2001; Lévesque et al., 2003; Ochsner *et al.,* 2002; Ochsner *et al.,* 2004; Phan *et al*., 2004; Kim and Hamann, 2007; Delgado *et al.,* 2008). The dlPFC is thought to contribute to cognitive control processes to regulate emotions (Ochsner and Gross, 2005; Delgado *et al.,* 2008). The dlPFC may indirectly control emotions via projections to the ventromedial or lateral prefrontal cortices, regions that can inhibit activity in regions critical to the behavioural expression of emotions, such as the amygdala (Ochsner and Gross, 2005; Delgado *et al.,* 2008; Buhle *et al.,* 2014). Furthermore, a recent meta-analyses of neuroimaging studies of fear conditioning in humans revealed dlPFC activation during the extinction of conditioned threat (Fullana *et al.,* 2015), perhaps indicating that the dlPFC is also involved in some aspects of threat response reduction. As discriminatory threat learning typically also involves reduction of threat responses to a safe stimulus, it could be that the dlPFC is also involved in acquisition of discriminatory threat responses via cognitive regulation of threat responses to a safe stimulus. Although neuroimaging studies have provided correlational evidence for a role of the dlPFC in cognitive regulation of emotions such as fear, causal evidence is lacking.

Studying patients with brain lesions can reveal whether brain structures are critical to emotional learning and regulation. Such lesions studies have revealed the necessity of the amygdala for conditioned threat responses, the hippocampus for explicit emotional memory and the medial prefrontal cortex for the integration between emotion and cognition (Bechara *et al.,* 1995; LaBar *et al.,* 1995; Bechara *et al.,* 1999; Funayama *et al.,* 2001; Gläscher *et al.,* 2012; Klumpers *et al.,* 2015b). However, whether damage of the dlPFC would be associated with impaired cognitive regulation of emotions is still unknown.

**Fig. 1:**
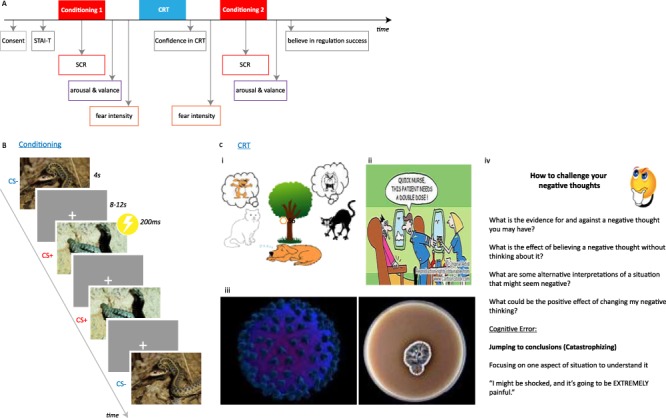
Study design. (**A**) Timeline of the study design and obtained measures. (**B**) In the threat-conditioning task, participants were presented with images of snakes or spiders and one of the two images was partially reinforced by electrical shock (CS+) while the other was not (CS-) allowing assessment of excitatory threat learning and inhibitory threat regulation, respectively. (**C**) During CRT, participants were shown images and these were used to explain that thoughts can drive emotions and that changing one’s thoughts can change one’s feelings. (i) The cartoon used to explain that thoughts influence emotions. Even though both cats are in the same situation, their thoughts about the dog result in different emotional responses. (ii) The cartoon used to illustrate that experience in a situation can influence one’s thoughts, which influences one’s emotional responses. The man on the left is an infrequent flier and believes the plane may crash so he is extremely nervous and uneasy. The woman on the right is a flight attendant used to flying and is thus relatively calm. (iii) The cartoon is used to explain that new information can change the way we think and change the way we feel. The image on the left may be seen as a pleasant picture but might be viewed as unpleasant when informed that it depicts HIV. Then the picture on the right may be seen as unpleasant but might be viewed as pleasant once informed that it depicts penicillin. (iv) This image was used to prompt participants to come up with alternative thoughts about the image paired with shock and to explain ‘catastrophizing’, i.e. focusing on one negative aspect of a situation rather than the whole situation. Following the laboratory version of CRT patients’ and healthy control participants’ ability to regulate their emotions were assessed with subjective measures of emotion and of threat responses during second conditioning session.

The objective of the present study was to determine if the prefrontal cortex is critical to the acquisition and/or cognitive regulation of subjective fear and autonomic responses to threat. We tested patients with prefrontal cortical lesions and healthy control participants’ ability to use a laboratory version of cognitive regulation treatment (Shurick *et al.,* 2012) to regulate subjective fear and autonomic responses within a threat-conditioning paradigm (Figure 1). Briefly, participants underwent a threat-conditioning session. Next, participants received cognitive regulation training (CRT) during which we emphasized the link between thoughts and emotions, encouraging participants to reimagine the association between a threat-conditioned cue and an aversive outcome (mild electrical shock) in a more positive manner. We then assessed participants’ re-evaluation of subjective emotions and ability to mitigate autonomic responses to threat during a second threat-conditioning session. A previous study concluded that the induction of stress, which presumably impaired prefrontal cortical functioning (Arnsten, 2009), impaired people’s ability to regulate conditioned autonomic responses to threat, as well as subjective fear, following CRT (Raio *et al.,* 2013). We therefore hypothesized that patients with dorsolateral prefrontal lesions would show impaired cognitive regulation following CRT.

## Materials and methods

### Participants

We were able to recruit six patients with lesions spanning the dlPFC (including, but not limited to, lesions of BA9 and/or 46) from the Patient Registry for the Study of Perception, Emotion, and Cognition (PROSPEC) at NYU and 19 control participants [patients: 2 females, 4 males, age: 42.83 ± 6.52 (25–69), FSIQ: 101.7 ± 4.17 (82–112); controls: 8 females, 11 males, age: 37.11 ± 3.17 (22–70), FSIQ: 103.14 ± 3.36 (75–123)]. Given the limited number of patients we were able to include, results of this study should be interpreted with caution and will require replication with larger sample sizes in future studies. Patients had lesions of the prefrontal cortex due to surgical resection as treatment for pathology including gliomas (*N* = 3), meningioma (*N* = 1), epilepsy (*N* = 1) or glioma-causing epilepsy (*N* = 1). Prior to surgery, areas adjacent to BA 9 and 46 (dlPFC) had been functionally mapped before resection, as to spare eloquent cortex. Note that lesion were not limited to the dlPFC as the need for resection can hardly ever be that locally restricted (see Results). Control participants were free of known neurological history and all participants, patients and controls, had no known history of major psychiatric conditions and had normal or corrected-to-normal vision and normal hearing. Intelligence was measured by the Wechsler Adult Intelligence Scale-Fourth Edition (WAIS-IV), during administration of a larger battery of neuropsychological tests during the comprehensive screening procedures for PROSPEC inclusion. The Full Scale Intelligence Quotient (FSIQ) from the WAIS-IV was the single metric used to determine general intelligence for the patients and control subjects. Four control participants did not undergo intelligence assessment at the time of the study, thus they were not included in the FSIQ analysis. Patients also completed the Boston Naming Test on which they were asked to name drawn objects; a Verbal Fluency Test on which they were asked to name as many animals as possible in 60 seconds; a Phonemic Fluency Test on which they were asked to name as many words as they could think of beginning with a specific letter in three rounds for three different letters; and a rapid Stroop Word Reading Test on which they were asked to read columns of color names as fast as they could in 45 seconds, to ensure normal verbal fluency and safeguard against aphasia being a confounding factor. Participants had educational backgrounds spanning from incomplete high school education to a graduate level degrees. To safeguard against potential group differences on follow-up and post-hoc tests being driven by differences in statistical power between groups due to differences in group size, we also selected six control participants as ‘matched-controls’ based on most similar age, sex and FSIQ score to the patients for additional comparisons. The study was approved by the University Committee on Activities Involving Human Subjects at New York University. All participants provided written informed consent. All methods were carried out in accordance with the Declaration of Helsinki.

### Structural imaging and lesion mapping

Three-dimension (3D) magnetization-prepared rapid gradient-echo (MP-RAGE) T1-weighted sequences were obtained for each patient subject. Patients were excluded if there was evidence of diffuse atrophy on magnetic resonance images (MRI). Imaging procedures were performed at the NYU Center for Brain Imaging by a board certified neuropsychologist with assistance from a trained MRI technician and physicist on a 3-Tesla Siemens Allegra head-only MR scanner when post-surgical scans were not available from the Department of Radiology at the NYU School of Medicine. Medical Center scans were obtained using 1.5 or 3-Tesla Siemens full-body MR scanners. Image acquisitions included a conventional three-plane localizer and two T1-weighted gradient-echo sequence (MP-RAGE) volumes (TE = 3.25 ms, TR = 2530 ms, TI = 1.100 ms, flip angle = 7°, field of view = 256 mm, voxel size = 1×1×1.33 mm). Acquisition parameters were optimized for increased gray/white matter image contrast.

**Fig. 2:**
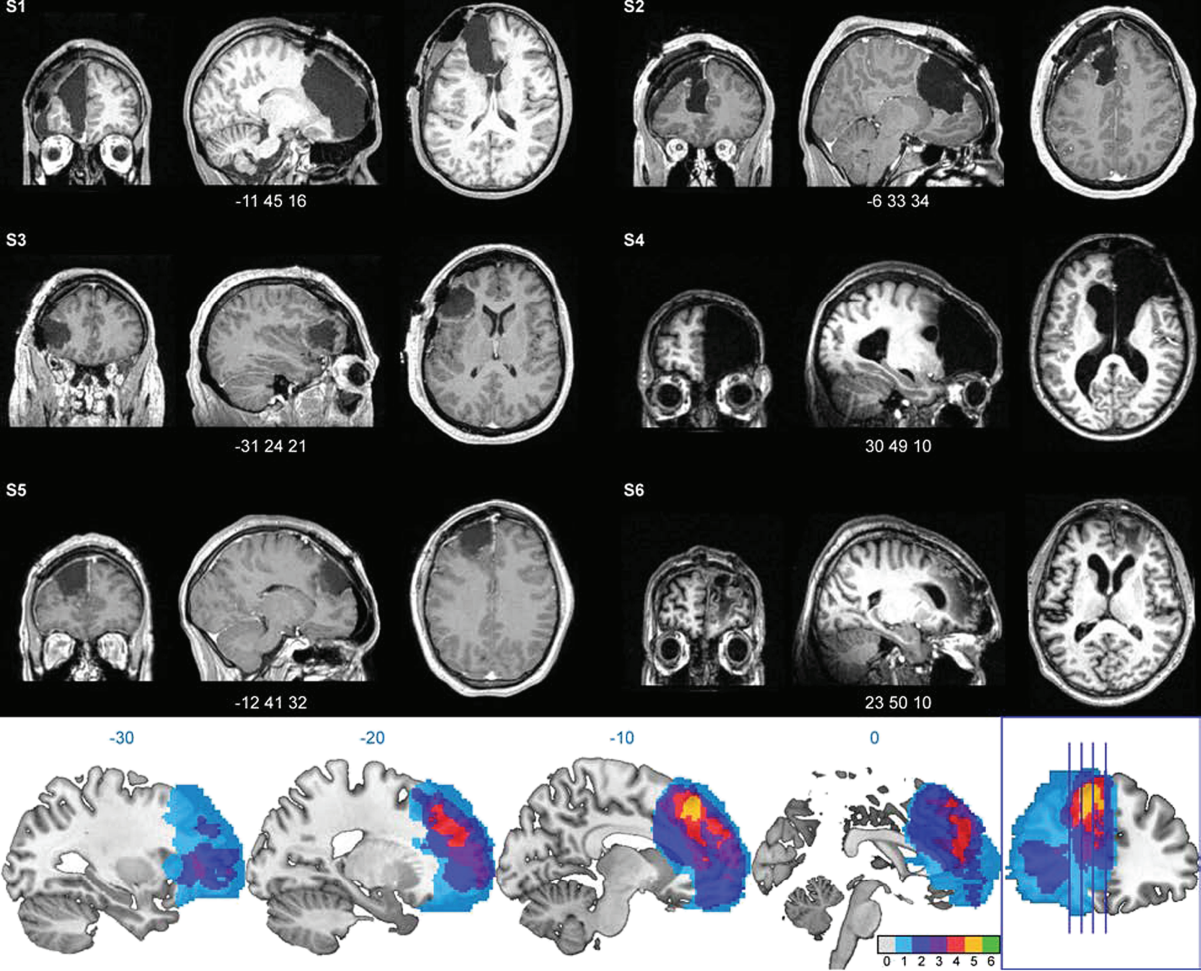
Structural MRIs of individual patients and lesion overlap. Top: spatially normalized structural MRIs of individual patients (S1–6) revealing extend of lesions. Images are displayed at the centre of the lesion (MNI coordinates below). Bottom: depiction lesion overlap across patients (colour represent number of patients with lesion at a particular voxel [0–6], note that lesion maps of patients with right-sided lesion have been left-right flipped to better depict commonly affected brain region).

Anatomical T1-weighted magnetic resonance images of patients obtained from PROSPEC were spatially normalized into a common stereotactic space (MNI 152 T1-template, 12 parameter affine linear transformation and nonlinear transformation at regularization = 1) using SPM12 (Wellcome Trust Centre for Neuroimaging, London, UK; http://www.fil.ion.ucl.ac.uk/spm). This consisted of a two-step procedure: first, using MRIcron (http://www.mccauslandcenter.sc.edu/mricro/mricron/), a mask was drawn over the lesion and any craniotomy defect to prevent bias in the transformation, then masked voxels were assigned a weight of ‘0’ and ignored during 12° affine transformation of the lesioned brain to the standard MNI 1 mm reference volume. The second procedure required the lesions to be manually traced on individual slices of the patients’ brains overlaid on the standard MNI brain template, requiring review in all three planes for accuracy. This tracing procedure produced a 3D volume with ‘1’ indicating the presence of the lesion and ‘0’ the presence of normal tissue. Most of the patients’ lesions had margins, which appeared readily visible on T1-weighted MRI images. In instances where there was uncertainty regarding the lesion margins, the treating neurosurgeon(s) and/or neuroradiologist was consulted. Finally, individual lesion maps were overlaid using MRIcon (see Figure 2).

### Threat conditioning

The Pavlovian threat-conditioning task (Figure 1B) was identical to a previous publication (Raio *et al.,* 2013). Briefly, each conditioning task comprised of two conditioned stimuli (CS+, CS-). The CSs were either two images of snakes or spiders. If participants scored higher on the Snake Anxiety Questionnaire than the Spider Phobia Questionnaire (Klorman *et al.,* 1974), the images of spiders were used and vice versa. Note, if patients reached phobic criteria, i.e. a score >15 out of 30, they were excluded in the study, which turned out not to be necessary for any of the participants. The CSs were presented for 4 s on a grey background with an 8–12 s inter-trial interval with a fixation cross on a blank screen. The unconditioned stimulus (US) was a mild electrical shock (200 millisecond asymmetric biphasic pulse with a width of 250 μs at 150 Hz) delivered to the right wrist using disposable pre-gelled electrodes connected to a Grass Medical Instruments stimulator (Grass Medical Instruments stimulator, Warwick, RI) that co-terminated with the CS+. There were eight presentations of the CS+ that co-terminated with the US intermixed with 17 CS+ (~33% reinforcement rate) and 17 CS- trials that did not co-terminate with the US. The presentation order of CSs was pseudo-randomized so that no more than three trials of the same type occurred in a row and the first trial was always a reinforced CS+.

### Cognitive regulation treatment

We used a laboratory version of cognitive regulation treatment (Figure 1C), as described previously (Shurick *et al.,* 2012; Raio *et al.,* 2013). Briefly, participants are presented with cartoon images that aid the experimenters’ explanation that: (i) changing thoughts about a situation can regulate emotional responses, (ii) differences in experience can regulate emotional feelings about a situation, that (iii) adding new information can regulate thoughts and emotional feelings about a situation and (iv) explains the concept of ‘catastrophizing’, i.e. that irrational thoughts can make us believe that a situation is worse than it actually is.

### Skin conductance responses

Galvanic skin conductance was measured from the hypothenar eminence of the palmar surface of the non-dominant hand with pre-gelled snap electrodes (BIOPAC EL509) and continuously recorded at 200 samples per second using a BIOPAC MP-100 System (Goleta, CA). Skin conductance responses (SCRs) were assessed using an in-house analysis program written in Matlab (the MathWorks) as described previously (Dunsmoor *et al.,* 2015). Responses were determined for each trial if the trough-to-peak deflection occurred 0.5–3 s following CS onset, lasted between 0.5 and 5.0 s and was greater than 0.02 μSiemens. Responses that did not meet these criteria were scored as zero. The raw SCRs were square root transformed and analyses restricted to non-reinforced trials only, in accordance with previous literature (Lykken and Venables, 1971; Schiller *et al.,* 2008; Dunsmoor *et al.,* 2015; Klumpers *et al.,* 2015a; Kroes *et al.,* 2015; Kroes *et al.,* 2017a, Kroes *et al.,* 2017b). Mean scores for the early (first half of the trials of a task) and late (second half of the trials of a task) phase were calculated for each task.

### Subjective emotion measures

Subjective fear was measured for the CS+ by participants reporting three emotions to the question: ‘*What are your emotions regarding this image or, in other words, when you see this image, how does it make you feel?*’ Next, the intensity of each individual emotional word was rated by answering the question ‘*Can you please rate that feeling on a scale of 1–10, 1 being the least intense and 10 being the most intense?’* Intensity of specific thoughts was measured by participants answering the question, ‘*What are your thoughts when you see this image? How strongly do you believe in these thoughts on a scale from 1–10, 10 being the most strong?*’ The emotion words were categorized as fear, disgust, sadness, anger, surprise or neutral independently by two raters blinded to the participants identity upon completion of the study (inter-rater reliability across all words = 0.92 and limited to words rated as fear by either rater 0.95, disagreement were resolved via discussion between the raters). As our interest here was in emotional regulation to threatening stimuli, we only analysed emotions categorized as fear. Subjective fear was calculated as the cumulative intensity rating of the reported fear words divided by the maximum possible cumulative rating score. Thus, for example, for two reported fear words the intensity rating of both words, e.g. 5 + 6 = 11, was divided by the maximum possible cumulative score, i.e. 3^*^10 = 30, resulting in a normalized score, e.g. 11/30 = 0.367. Subjective valence and arousal was measured to both CSs using a Manikin Scale ranging from 1 to 9 (valence: negative-positive; arousal: calm-excited) (Bradley and Lang, 1994).

### Subjective CRT success

Subjective success of CRT was indexed by rating ‘*how successful participants felt they were at changing their thoughts and feelings towards the image paired with shock and the shock itself*’ on a 0–10 scale, with 0 representing no success and 10 representing complete success.

### State and Trait Anxiety Inventory

The State and Trait Anxiety Inventory (STAI-T) measured trait anxiety levels (Spielberger *et al.,* 1983).

### Procedures

Upon arrival, participants were informed that they would participate in a study on emotional reactions, be presented with pictures that might be paired with a mild electrical shock, and would be asked to discuss their thoughts and feelings. They signed the consent form and completed the STAI-T. Next, SCR and shock electrodes were attached. Shocks were calibrated using an ascending staircase procedure starting with a low voltage setting near a perceptible threshold and increasing to a level deemed ‘maximally uncomfortable but not painful’ by the participant, in keeping with previous threat-conditioning protocols (Schiller *et al.,* 2008; Klumpers *et al.,* 2015b; Kroes *et al.,* 2015; Dunsmoor et al., 2017; Kroes *et al.,* 2017a; Kroes *et al.,* 2017b). Hereafter, the conditioning task was conducted during which SCRs were recorded and after which subjective arousal and valence ratings were obtained. Next, electrodes were removed. Participants were instructed that they would discuss their thoughts and emotions regarding the experiment and would be shown images to explain the relationship between thoughts and emotions. We told them that ‘emotion’ was defined as a specific feeling they experienced that could be described by a single word, while a ‘thought’ was something more elaborate that involved an opinion, judgement or description of the situation. Then, participants were shown an image of the CS+ and listed three emotion words associated with the image and their intensity. They then underwent CRT where the experimenter emphasized the link between thoughts and emotions. We then asked them to create alternative more positive thoughts about the CS+ that were not centred on the association of the image with the shock or that the image might look frightening. Participants described their alternative thoughts and rated how confident they were at using these alternative thoughts to regulate their emotional responses. Then they re-rated the intensity of the previously listed emotional words and indicated how much they still believed in their original thoughts. Participants were told they would engage in an identical conditioning task and encouraged to use new thoughts to regulate emotional responses during the task. Electrodes were reattached and the conditioning task commenced during which SCRs were again recorded. Participants completed subjective arousal and valence ratings and then rated whether they believed that they were successful in changing their thoughts and feelings.

### Statistics

To compare acquisition of discriminative threat learning, we subjected the average SCR to threat (CS+) and safety (CS-) cues during the first half of the trials (early phase) and the second half of the trials (late phase) of the first conditioning session to a group (patient, control) × phase (early, late) × CStype (CS+, CS-) 2×2×2 repeated measures analyses of variance (rmANOVA). Valence and arousal and fear word intensity scores provided following the first conditioning session were subjected to group (patient, control) × CStype (CS+, CS-) 2×2 rmANOVA.

To assess cognitive regulation following the laboratory version of CRT, fear word intensity scores were subjected to a group (patient, control) × time (before, after CRT) 2×2 rmANOVA. Valence, arousal and SCR scores were subjected to a group (patient, control) × time (before, after CRT) × CStype (CS+, CS-) 2×2×2 rmANOVA. We compared SCRs during the second half of the first conditioning session (before CRT) with response during the first half of the second conditioning session (after CRT).

Statistics were Greenhouse-Geisser or Huynh-Feldt corrected for non-sphericity when appropriate. Significant findings from ANOVAs were followed up by paired and independent samples *t*-tests. As we were interested in group differences in cognitive regulation following CRT, we planned comparisons between groups on the change in critical variables from before to after CRT. Relevant mean ± standard error of mean (SEM) values are provided in figure legends.

**Table 1:** Number of classified emotion words produced by each group after conditioning prior to CRT

	Fear	Anger	Neutral	Disgust	Surprise	Happiness	Sadness	Total
Controls	30	9	4	3	3	1	1	51
Patients	6	4	5	3	0	0	0	18
total	36	13	9	6	3	1	1	69

**Fig. 3:**
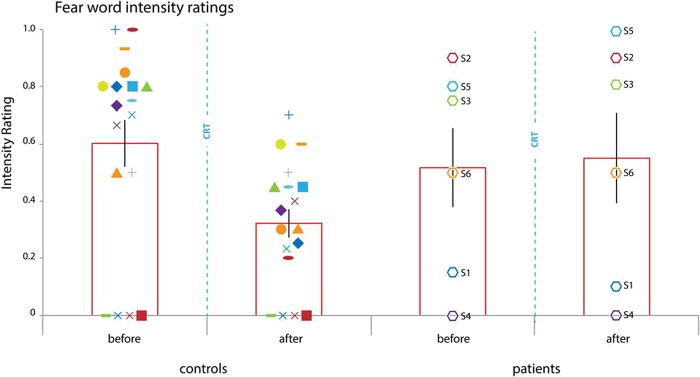
Fear word intensity ratings. Y-axis reflects normalized fear intensity rating. Participants could list maximally three fear words and give all of them a maximum intensity rating of 10 resulting in a cumulative maximum score of 3^*^10 = 30. Therefore, all cumulative intensity scores were normalized by division by 30, for example, if a participant listed two fear words of which one was rated at intensity 7 and the other at intensity 8, then the intensity rating was calculated as: (7 + 8)/30 = 0.5. Patients and controls acquired conditioned subjective fear word ratings. Control participants showed a drop in fear word intensity ratings following CRT whereas prefrontal lesion patients did not. (mean ± SEM: control before CRT: 0.644 ± 0.077; control after CRT: 0.330 ± 0.050; patients before CRT: 0.517 ± 0.151; patients after CRT: 0.550 ± 0.173). Error bars reflect SEM^*^ = *P* < 0.05. Octagon symbols represent PFC lesion patients and numbers are identical to those listed in Figure 2 (S1 = dark blue, S2 = red, S3 = green, S4 = purple, S5 = light blue, S6 = orange).

## Results

### Participants

Patients and controls did not differ in regards to intellectual functioning (WAIS-IV) FSIQ [t(18) = 0.338, *P* = 0.719, patients: 101.167±4.17; controls: 103.14±3.36] or any of the four primary WAIS-IV indices Verbal Comprehension Index [t(18) = −0.124, *P* = 0.902, patients: 112.83±5.00; controls: 112.071±3.39], Perceptual Reasoning Index [t(18) = 0.145, *P* = 0.886, patients: 97.67±3.45; controls: 98.429±3.09], Working Memory Index [t(18) = 0.241, *P* = 0.812, patients: 99.67±6.48; controls: 101.29±3.43], Processing Speed Index [t(18) = 1.349, *P* = 0.194, patients: 94.17±6.03; controls: 103.500±3.73] or self-ratings for trait anxiety (STAI-T) [t(22) = 0.834, *P* = 0.431, patients: 46±1.03; controls: 48.17±1.44]. One control participant had an FSIQ score more than two standard deviations above the mean and was excluded from analyses. Patients did not score different from the expected mean of the normed scores on the Boston Naming Test (z-score) (t(5) = −0.238, *P* = 0.821, mean:−0.0783, SEM: 0.329, 95% CI: [−0.923, 0.767]), Verbal Fluence Test (z-score) (t(5) = 1.515, *P* = 0.190, mean: 0.4700, SEM: 0.310, 95% CI: [−0.327, 1.267]), Phonemic Fluency Test (z-score) (t(5) = −0.674, *P* = 0.530, mean: −0.2600, SEM: 0.386, 95% CI: [−1.252, 0.732]) or Stroop Word Reading Test (T-score) (t(5) = 1.000, *P* = 0.363, mean: 52.333, SEM: 2.333, 95% CI: [46.335, 58.331]) and thus had no impairment in verbal fluency and were not aphasic. Two control participants had SCRs during conditioning more than two standard deviations above the mean and were excluded from SCR analyses.

All patients had lesions including the dlPFC (Figure 2). One patient had a left-sided lesion encompassing the orbitofrontal cortex, medial prefrontal cortex, anterior cingulate cortex and superior and medial prefrontal gyrus. A second patient had a left-sided lesion spanning the superior frontal gyrus, medial prefrontal cortex and anterior cingulate cortex. A third patient’s lesion centred on the left middle frontal gyrus. A fourth patient had a right-sided lesion of the orbitofrontal cortex, medial prefrontal cortex, anterior cingulate cortex and superior and medial prefrontal gyrus. A fifth patient’s lesion included the left superior frontal cortex and medial prefrontal cortex. The sixth patient had a right-sided lesion of the middle frontal gyrus, medial frontal gyrus and superior frontal gyrus. The greatest lesion overlap was observed for the superior frontal gyrus, superior medial gyrus and middle frontal gyrus corresponding to Brodmann’s areas 8 and 9.

### Confidence in ability to cognitively regulate emotions

Immediately after CRT, but before the second conditioning session, participants indicated that they were confident in using alternative thoughts to regulate their emotions [one sample *t*-test: t(23) = 17.161, *P* < 0.001; 7.8125±0.4553] and we found no difference between groups [independent samples *t*-test: t(22) = −1.088, *P* = 0.289; controls: 7.5278±0.5864; patients: 8.667±0.3333]. At the end of the experiment, i.e. after CRT and after the second conditioning session, we asked participants to estimate how effective they had been in regulating their emotions following CRT. An independent samples *t*-test revealed no differences between patients and controls [t(22) = −0.102, *P* = 0.920] and a one-sided *t*-tests (measures below zero were not possible) showed that all groups estimated that they had been able to regulate their emotions [controls: t(17) = 12.408, *P* < 0.001; patients: t(5) = 9.045, *P* < 0.001; matched-controls: t(5) = 5.000, *P* = 0.004]. Thus, both patients and controls indicated being confident that they could, and did, use CRT to regulate their emotions.

**Fig. 4:**
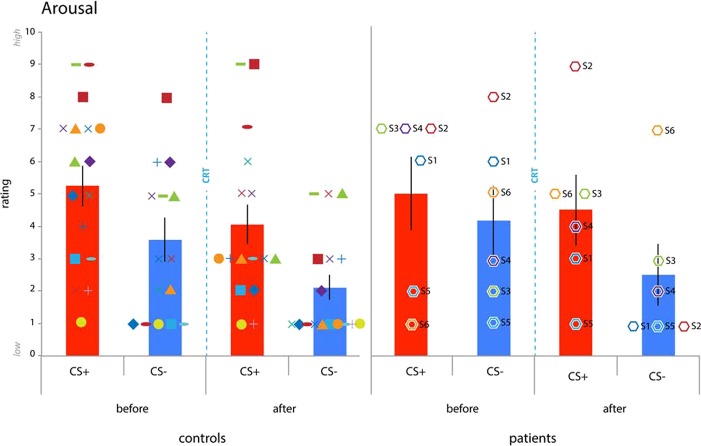
Arousal ratings. Patients and controls acquired differential conditioned arousal ratings. Following CRT, we found a reduction in arousal rating for the control participants but not for the PFC patients (mean ± SEM: controls before CS+: 5.2353 + −0.6215; before CS-: 3.5882 + −0.6755; after CS+: 4.0588 + −5.9699; after CS-: 2.1176 + −0.3824; before CS+: 5.000 + −1.1255; before CS-: 4.1667 + −1.0776; after CS+: 4.500 + −1.0878; after CS-: 2.500 + −0.9574). Error bars reflect SEM^*^ = *P* < 0.05. Next we explored possible group differences in regulation of arousal ratings following CRT. Exploratory analyses revealed reduced arousal ratings in the control group for the CS+ [t(17) = 4.777, *P* < 0.001] and CS- [t(17) = 2.976, *P* = 0.008] but not the patients [CS+: t(5) = 0.425, *P* = 0.688; CS-: t(5) = 1.147, *P* = 0.303]. Limiting the rmANOVA to patients and matched control also revealed a main effect of CStype [F(1,10) = 9.186, *P* = 0.013] with no other main effects or interactions. Exploratory analyses hint at a decrease in arousal rating for the matched-controls for the CS+ [t(5) = 3.464, *P* = 0.018] but not CS- [t(5) = 1.581, *P* = 0.175]. Thus, patients and controls acquired comparable differential conditioned arousal that decreased following CRT and exploratory analyses suggest that controls might have been able to cognitively regulate arousal for the CS+ following CRT but not patients. Octagon symbols represent PFC lesion patients and numbers are identical to those listed in Figure 2 (S1 = dark blue, S2 = red, S3 = green, S4 = purple, S5 = light blue, S6 = orange).

### Subjective fear ratings

Immediately before CRT, participants listed three emotional words they associated with the conditioning procedure. Both patients and controls predominantly reported words classified as ‘fear’ (Table 1). A Fisher’s exact test revealed no differences in reported emotion word categories between patients and controls (*P* = 0.126).

Participants rated the intensity of the emotional words they had associated with the conditioning procedure and re-rated the intensity of these same words after CRT (Figure 3). A group (patients, controls) × time (before, after CRT) revealed a group × time interaction [F(1,22) = 12.766, *P* = 0.022, η^2^ = 0.367], a main effect of time [F(1,22) = 8.345, *P* = 0.009, η^2^ = 0.275], but no main effect of group [F(1,22) = 56.175, *P* = 0.737]. A follow-up independent samples *t*-test on the subjective fear ratings after conditioning but before CRT revealed no differences in the acquisition of subjective fear between groups [t(22) = 0.428, *P* = 0.807].

Examining the effect of CRT and potential group differences, follow-up independent samples *t*-tests on change scores (pre-post intensity rating) revealed greater change in subjective fear ratings in the control than patient group [t(22) = −3.573, *P* = 0.002] where one-sample *t*-tests revealed a change in subjective fear in controls [t(16) = 4.879, *P* < 0.001] but not patients [t(5) = −0.933, *P* = 0.394]. Limiting the analyses to patients and matched-controls, a group (patients, controls) × time (before, after CRT) also revealed a group × time interaction [F(1,10) = 31.884, *P* < 0.001], a main effect of time [F(1,10) = 23.517, *P* = 0.001], but no main effect of group [F(1,10) = 0.258, *P* = 0.622]. Again, an independent samples *t*-test revealed a greater change in subjective fear ratings in the matched-control group compared to the patient group [t(10)=−5.647, *P* < 0.001], and so did a Mann–Whitney rank order test (*U* = 10, *P* = 0.003, two-tailed) which we ran to guard against the possibility that this group difference resulted from non-normal distributions in small samples. Thus, both groups displayed comparable subjective fear acquisition but where the control participants were able to cognitively regulate subjective fear immediately following CRT it appeared that the prefrontal patients were not.

### Arousal and valence ratings

Following the first and second conditioning session, participants provided subjective arousal and valence ratings for the threat (CS+) and safe stimulus (CS-) (Figure 4). Comparing subjective arousal ratings after the first conditioning session but before CRT with the ratings after CRT and after the second conditioning session with a group (patient, control) × time (before, after) × CStype (CS, CS-) revealed a main effect of time [F(1,22) = 13.071, *P* = 0.002, η^2^ = 0.373] and CStype [F(1,22) = 9.110, *P* = 0.006, η^2^ = 0.293] with no other main effects or interactions. A follow-up paired *t*-test on average arousal ratings for the CS+ and CS- following the first and second conditioning session revealed that participants rated the CS+ as more arousing than the CS- [t(23) = 3.939, *P* = 0.001. CS+: 9.7917**±**0.9421; CS-: 5.6667**±**0.7265], indicating successful acquisition of differential conditioned subjective arousal. A follow-up paired *t*-test on average arousal ratings across CS+ and CS- trials for the first and second conditioning session revealed greater arousal ratings for the conditioning session before CRT than after CRT [t(23) = 4.634, *P* < 0.001. Before: 9.2083±0.7801; after: 6.2500±0.6790]. Limiting analyses to patients and the matched-control group did not reveal any additional effects. Hence, both groups acquired discriminatory threat conditioned arousal ratings, provided reduced arousal ratings to both the CS+ and CS- following CRT, and we observed no differences between groups.

**Fig. 5:**
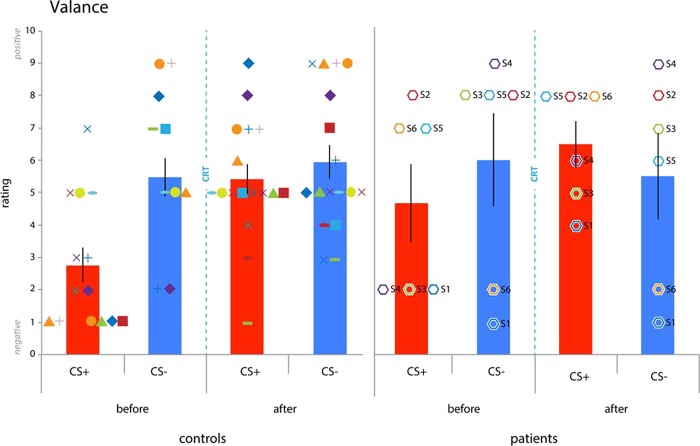
Valence ratings**.** Both patients and controls acquired differential conditioned valence ratings. Both groups appeared to be able to regulate valence following CRT (mean ± SEM: controls: before CS+: 2.765 + −0.5323; before CS-: 5.4706 + −0.5886; after CS+: 5.4118 + −0.4542; after CS-: 5.9412 + −0.5179; patients: before CS+: 4.6667 + −1.2019; before CS-: 6.0000 + −1.4376; after CS+: 6.500 + −0.7188; after CS-: 5.500 + −1.3354). Error bars reflect SEM^*^ = *P* < 0.05. Octagon symbols represent PFC lesion patients and numbers are identical to those listed in Figure 2 (S1 = dark blue, S2 = red, S3 = green, S4 = purple, S5 = light blue, S6 = orange).

Comparing the subjective valence ratings (Figure 5) after the first conditioning session but before CRT with the ratings after CRT and after the second conditioning session with a group (patient, control) × time (before, after) × CStype (CS+, CS-) revealed a time × CStype interaction [F(1,21) = 9.204, *P* = 0.006] with no other interaction or main effect. A group (patient, control) × CStype (CS+, CS-) ANOVA limited to subjective valence ratings obtained after the first conditioning session but before CRT revealed a main effect of CStype [F(1,22) = 6.392, *P* = 0.018, η^2^ = 0.229] with no other main effect or interaction. A follow-up paired samples *t*-test indicated that following conditioning participants rated the CS+ as more negative than the CS- [t(23)-3.577, *P* = 0.002. CS+: 3.000±0.507; CS-: 5.875±0.5592], indicating the successful acquisition of differential conditioned subjective valence across both groups and no differences between them. Next we assessed the effect of CRT on the regulation of subjective valence. A follow-up paired samples *t*-tests across groups revealed an increase in subjective valence ratings for the CS+ [t(23) = −4.479, *P* < 0.001] but no change for the CS- [t(23) = −0.301, *P* = 0.766] following CRT. Limiting analyses to patients and the matched-control group did not reveal any additional effects. Thus, both patients and controls acquired differential conditioned subjective valence and CRT increased subjective valence ratings for the CS+ in both groups.

**Fig. 6:**
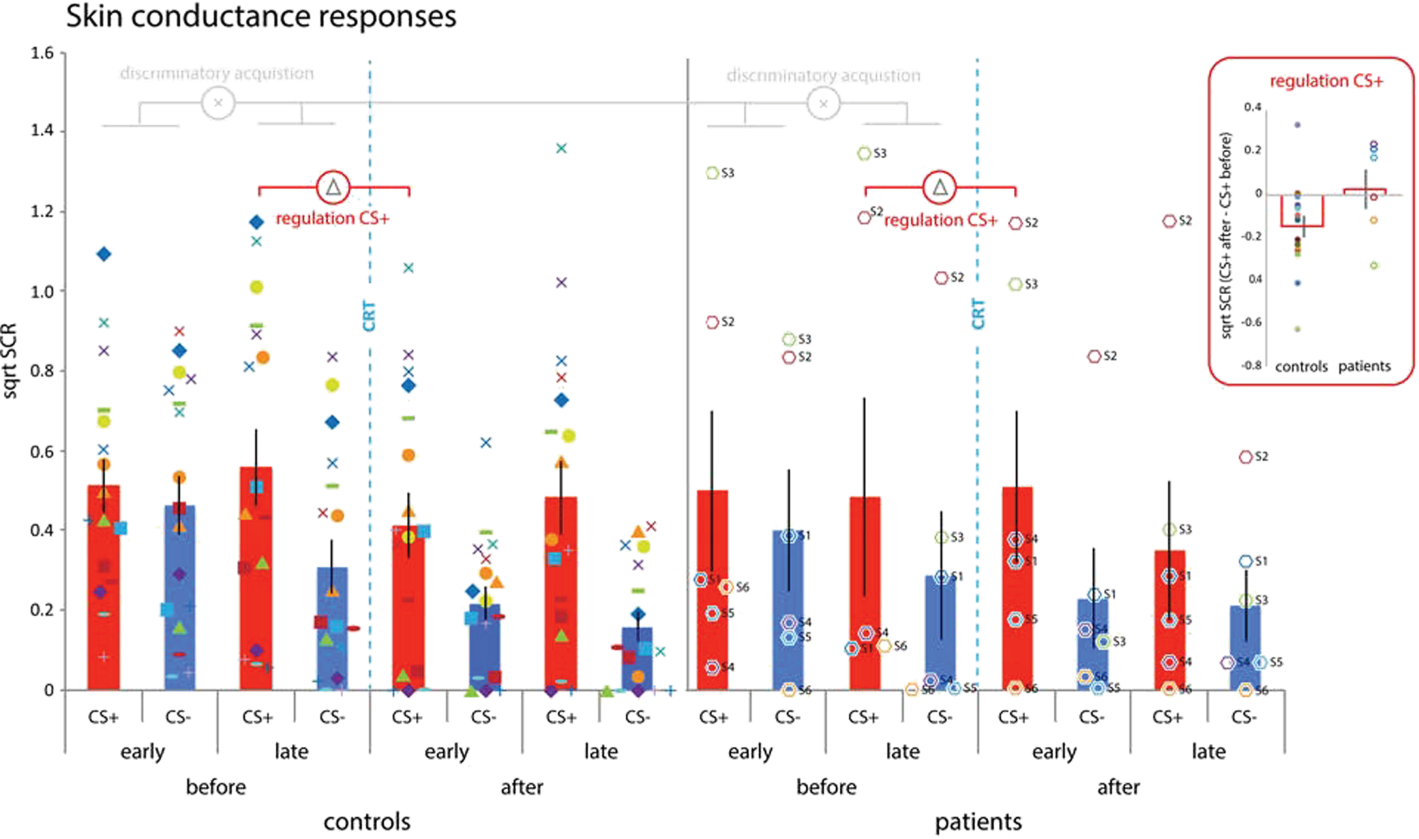
Skin conductance responses. Both patients and controls acquire differential threat conditioned SCRs and exhibit regulation of responses to the CS- during acquisition (mean ± SEM: controls: before early CS+: 0.5142 + −0.0703; before early CS-: 0.4354 + −0.0744; before late CS+: 0.5658 + −0.0991; before late CS-: 0.2997 + −0.0716; patients: before early CS+: 0.4993 + −0.2018; before early CS-: 0.3999 + −0.1537; before late CS+: 0.4825 + −0.2493; before late CS-: 0.2868 + −0.1627). Following CRT, exploratory analyses suggest that only the controls might have been capable to use CRT to regulate SCRs to the CS+ (mean ± SEM: after early CS+: 0.4166 + −0.0868; after early CS-: 0.2081 + −0.0437; patients: after early CS+: 0.5091 + −0.1927; after early CS-: 0.2287 + −0.1260). Inset: regulation score for CS+, i.e. difference in mean SCR responses to the CS+ from the late phase before CRT to the early phase after CRT. Error bars reflect SEM. Octagon symbols represent PFC lesion patients and numbers are identical to those listed in Figure 2 (S1 = dark blue, S2 = red, S3 = green, S4 = purple, S5 = light blue, S6 = orange).

### Skin conductance responses

We assessed discriminatory threat learning during the first conditioning session expressed as SCRs (Figure 6). A group (patient, control) × phase (early, late) × CStype (CS+, CS-) revealed a main effect of CStype [F(1,20) = 11.327, *P* = 0.003, η^2^ = 0.362] and a phase × CStype interaction [F(1,20) = 5.747, *P* = 0.026, η^2^ = 0.223] with no other main effects or interactions. Paired samples *t*-tests on the differences scores (CS+ - CS-) for the early and late phase indicated greater differential responses in the late than early phase [t(21) = −3.114, *P* = 0.005], that is driven by a decrease in responses to the CS- from the early phase to the late phase [t(21) = 3.495, *P* = 0.002] but no changes in responses to the CS+ [t(21) = −0.857, *P* = 0.401]. Hence, across patients and controls we found evidence for discriminatory threat condition SCRs that is driven by inhibitory regulation of responses to the safe stimulus, but we found no differences between groups.

Next we probed group difference in regulation of SCR following the laboratory version of CRT. A group (patient, control) × time (before, after CRT) × CStype (CS, CS-) revealed a main effect of CStype [F(1,20) = 13.318, P = 0.002, η^2^ = 0.400] and a main effect of time [F(1,20) = 5.754*, P* = 0.026, η^2^ = 0.223] with no other main effects or interactions. A follow-up paired samples *t*-test across conditioning sessions revealed greater average SCRs to the CS+ than CS- [t(21) = 4.471, *P* < 0.001. CS+: 0.4925±0.0851; CS-: 0.2550±−0.2457], indicating acquisition and maintenance of differential conditioned responses in both groups without evidence for differences between groups.

As Raio *et al.* (2013) suggested that CRT may specifically reduce SCR responses to the CS+, we ran an exploratory independent *t*-test on the change in SCR to the CS+ from before to after CRT and found a trend for differences SCR changes to the CS+ between groups [t(20) = 1.733, *P* = 0.098]. Follow-up tests revealed evidence for regulation in the control group [t(15) = 2.876, *P* = 0.012], but not the patient group [t(5) = −0.290, *P* = 0.783]. For the matched-controls, we also observed a decrease in SCR to the CS+ following CRT [t(5) = 3.343, *P* = 0.020]. None of the groups showed changes in regulation of SCR to the CS- (*P* > 0.1). These exploratory results thus hint that control participants might have been able utilize CRT to regulate SCRs to the CS+ while patients might not have been able to.

## Discussion

Our results suggest that patients with dlPFC lesions are capable of discriminatory threat learning but might be impaired in the cognitive regulation of subjective fear. Following threat conditioning both patients with lesions including the dlPFC and control participants exhibited equal acquisition of a threat memory as assessed by measures of subjective fear, arousal and valence and autonomic responses. Both lesion patients and controls were confident that they could cognitively regulate their emotions following a laboratory version of CRT. However, following CRT, patients did not exhibit a reduction in subjective fear, in contrast to control participants. CRT also resulted in a non-differential decrease in subjective arousal (i.e. arousal decreased for both the threatening and safe cue) and CRT rendered valence rating of the threatening cue more positive in both the control and patient groups with no differences between them. Following CRT, we observed a similar decrease in SCR to both the CS+ and CS- in both groups. Exploratory analyses suggest that patient might not have been able to regulate SCRs to the CS+ following CRT whereas controls could. Hence, despite the inclusion of a limited number of lesion patients, our results suggest that patients with prefrontal lesions are capable of discriminatory threat learning but appear impaired in the cognitive regulation of subjective fear, and potentially autonomic responses, to threat following a laboratory version of CRT.

There is extensive laboratory research on the cognitive regulation of subjective and physiological components of emotion (Gross and Levenson, 1997; Ochsner and Gross, 2005). The current data suggests that the dlPFC may be critical for the cognitive regulation of subjective fear, supporting previous publications that reported correlations between cognitive regulation of emotions and dlPFC activity (Ochsner *et al.,* 2002; Delgado *et al.,* 2008; Ochsner *et al.,* 2012). These studies suggested that cognitive regulation activates the dlPFC which down-regulates amygdala responses potentially via the ventromedial or lateral prefrontal cortex (Ochsner and Gross, 2005; Delgado *et al.,* 2008; Ochsner *et al.,* 2012). The dlPFC has also been implicated in working memory and attention modulation, which might be critical functions to support cognitive regulation. Yet, we observed differences in cognitive regulation in the absence of differences in IQ or working memory performance between controls and patients. This suggests that impairments in cognitive regulation could exist beyond detectable impairments in other cognitive functioning. Similarly, the specific impairment of cognitive regulation of subjective fear in dlPFC lesion patients was observed even though patients were free of psychiatric history and did not exhibit abnormal anxiety scores, indicating that our findings do not result from unspecific changes in mood or psychiatric functioning.

A limitation to our study is that most of the patients in our study had lesions that were not isolated to the dlPFC, but included lesion of the ventral PFC and in some cases also extended to the vmPFC. It is therefore possible that the cognitive regulation impairment that we observed is, at least in part, due to damage in prefrontal regions beyond the dlPFC. Due to the limited number of patients, lesion-to-symptom mapping was not possible to resolve this matter. Regardless, impaired cognitive regulation was also observed in patients with lesions that did not span into the ventromedial or ventrolateral prefrontal cortex (including for the patient with a lesion limited to the dlPFC). Furthermore, all patients showed a reduction of threat-related SCR responses to the safe CS- stimulus over the course of discriminatory threat learning. This reduction in threat responses is akin to extinction learning which is associated with the ventromedial prefrontal cortex (Fullana *et al.,* 2015). This could suggest that ventromedial prefrontal cortex functioning was unimpaired across our patient group.

Some (Bechara *et al*., 1995; Bechara *et al.,* 1999; Funayama *et al.,* 2001; Gläscher *et al.,* 2012; Mackey *et al.,* 2016), but not all (LaBar *et al.,* 1995; Strange *et al.,* 2003; Bach *et al.,* 2015; Klumpers *et al*., 2015b; Korn *et al.,* 2017; Terburg *et al.,* 2018), studies with lesion patients have included an additional patient group with lesions in a different region than that of interest. The inclusion of an additional lesion group can (i) serve as a control for unspecific effects of brain lesions on general functioning and (ii) serve to show double dissociations in lesion site and functional impairment. We did not include a lesion control group as we were not sure what lesion site would be an appropriate control for our study and because we were specifically interested in the effect of dlPFC lesions on discriminatory threat learning and cognitive regulation and not in search of double dissociations. This does, however, mean that we cannot exclude the possibility that the effects we report might be due to unspecific effects of brain lesions, disease or medication. Yet, the lesion patients in our study had no functional impairment on a host of other measures including intellectual functioning, verbal fluency and discriminatory threat acquisition. Thus, the impairment we observe seems limited to cognitive regulation of subjective fear, broadly in line with previous neuroimaging literature in this field. Taken together, we think that the dlPFC is the most likely candidate for the observed impairment in top-down emotion regulation even though it may exert its effect via modification of large-scale neural network functioning.

An interesting question is whether the dlPFC plays a role in cognitive regulation of only subjective emotions or also autonomic emotional responses. Our results suggest that patients with dlPFC lesions are impaired in cognitive regulation of subjective fear but we only found weak evidence for an impairment in cognitive regulation of autonomic SCRs to threat. Previous publications reported that CRT resulted in the regulation of differential threat-conditioned SCRs (Shurick *et al.,* 2012) or in a reduction of SCRs to the threatening CS+ stimulus only (Raio *et al.,* 2013). We found a reduction in SCRs to both the threatening CS+ and safe CS- stimulus following CRT. Considering the psychophysiology of SCRs, this could reflect cognitive regulation of SCR to both stimuli, physiological habituation or both. Additionally, it is important to note that the effect of CRT on autonomic responses was measured during a second conditioning session in which participants continued to receive electrical shocks. Similarly, valence and arousal ratings were also obtained after the second conditioning session. In the presence of threat, it is adaptive to exhibit some level of autonomic defensive responses. Future studies on cognitive threat regulation, per se, should focus on the effect of CRT on conditioned defensive responses during extinction, where the regulation of defensive responses would be adaptive. This may also help elucidate whether the preliminary evidence for impaired regulation of SCRs to the CS+ in dlPFC patients truly reflects impaired cognitive regulation of autonomic responses to threat.

We were also interested in the role of the dlPFC in discriminatory threat learning. A recent meta-analyses of neuroimaging studies of fear conditioning in humans revealed dlPFC activation during the extinction of conditioned threat (Fullana *et al.,* 2015), perhaps indicating that this region is involved in some aspects of threat inhibition. Particularly, healthy human populations typically have little trouble behaviourally discriminating between the CS+ and CS-, such that defensive responses are heightened on CS+ compared to CS- trials. In other words, healthy populations are capable of acquiring conditioned responses to a threat cue while simultaneously inhibiting emotional responses to a safety cue. In contrast, people with stress or anxiety disorders often exhibit intact acquisition of conditioned threat responses but impaired discrimination, as evidenced by a failure to inhibit threat responses to the learned safety cue (e.g. the CS-) or harmless stimuli that are physically similar to the CS+, referred to as over-generalization (Davis *et al*., 2000; Orr *et al.,* 2000; Lissek *et al.*, 2005; Jovanovic *et al.*, 2010). One possible mechanism for threat discrimination and inhibition in humans is top-down cognitive regulation of emotional responses, potentially by the dlPFC. However, we found no evidence for necessity of the dlPFC in the inhibition of autonomic threat responses to the CS- during threat conditioning to a perceptually similar CS+. Impaired top-down regulation by the dlPFC of emotional responses to safe stimuli may thus not explain the over-generalization of threat responses typically observed in patients with stress- and anxiety-related disorders (Davis *et al*., 2000; Orr *et al.*, 2000; Lissek *et al.*, 2005; Jovanovic *et al.*, 2010). Hence, threat over-generalization is likely mediated by aberrant neural processes in regions associated with bottom-up forms of emotion regulation, e.g. the ventromedial prefrontal cortex (Fullana *et al.,* 2015).

A limitation of the present study is that we were able to recruit only six patients with dlPFC lesions. To guard against potential differences in power between groups in explaining group differences, we also included analyses restricted to patients and six matched-control participants. Nonetheless, due to the fairly low sample size, generalizing from these results should be done with caution and result should be confirmed by replication studies with larger sample sizes.

Regardless of these studies limitations, our results suggest that the dlPFC may play a critical role in the cognitive regulation of subjective fear. The dlPFC was, however, not necessary for behavioural threat discrimination between a learned threat and a perceptually similar non-threat stimulus. The ability to cognitively regulate emotions predicts sensitivity to affective disorders and is a critical part of CBT (Cisler *et al.*, 2010; Beck and Dozois, 2011). Our results thus suggest that structural or functional impairments of prefrontal cortical functioning (e.g. via lesions or stress) may increase risk of affective disorders via an impairment of cognitive regulation but not via over-generalization of emotional responses. In turn, enhancement of prefrontal cortical functioning might be utilized to improve CBT treatment outcomes. Furthering understanding of the neural mechanisms of emotion regulation may thus change thoughts and emotions in clinical populations.
